# Cellular cooperation with shift updating and repulsion

**DOI:** 10.1038/srep17147

**Published:** 2015-11-25

**Authors:** Andreas Pavlogiannis, Krishnendu Chatterjee, Ben Adlam, Martin A. Nowak

**Affiliations:** 1 IST Austria, Klosterneuburg, A-3400, Austria; 2 Program for Evolutionary Dynamics, Department of Organismic and Evolutionary Biology, Department of Mathematics, Harvard University, Cambridge, MA 02138, USA

## Abstract

Population structure can facilitate evolution of cooperation. In a structured population, cooperators can form clusters which resist exploitation by defectors. Recently, it was observed that a shift update rule is an extremely strong amplifier of cooperation in a one dimensional spatial model. For the shift update rule, an individual is chosen for reproduction proportional to fecundity; the offspring is placed next to the parent; a random individual dies. Subsequently, the population is rearranged (shifted) until all individual cells are again evenly spaced out. For large population size and a one dimensional population structure, the shift update rule favors cooperation for any benefit-to-cost ratio greater than one. But every attempt to generalize shift updating to higher dimensions while maintaining its strong effect has failed. The reason is that in two dimensions the clusters are fragmented by the movements caused by rearranging the cells. Here we introduce the natural phenomenon of a repulsive force between cells of different types. After a birth and death event, the cells are being rearranged minimizing the overall energy expenditure. If the repulsive force is sufficiently high, shift becomes a strong promoter of cooperation in two dimensions.

Cooperation is a hallmark of living systems. Although the primary principle of biological evolution is natural selection, cooperation is ubiquitous in nature and is instrumental in generating higher levels of biological organization^1^,^2^. Cooperation can occur between replicating molecules, viruses, cells, multicellular organisms and people. Cooperation is usually opposed by natural selection unless a mechanism for evolution of cooperation is at work^3^. One such mechanism is spatial selection operating in structured populations^4^,^5^,^6^,^7^. Certain population structures enable cooperators to form clusters which can prevail in competition with defectors^8^,^9^,^10^. Here we study a mechanism facilitating cooperation among cells in a two-dimensional spatial model.

It is well known that population structure can change the outcome of evolutionary and ecological processes^8^,^11^,^12^,^13^,^14^,^15^,^16^,^17^,^18^,^19^,^20^. For example, deterministic evolutionary dynamics can lead to spatial chaos and emergence of cooperation on regular grids^8^. Evolutionary graph theory is a generalization of this approach to stochastic dynamics and any population structure^21^,^22^,^23^,^24^. The individuals of a population occupy the vertices of a graph. The links determine interaction and competition. There can either be two different graphs for interaction and competition^25^ or a single graph for both processes. The graph can be constant or changing under evolutionary updating^26^,^27^,^28^,^29^. Certain graphs enhance^30^ or suppress^31^ natural selection. Even the molecular clock of neutral evolution can depend on the graph structure^32^. Space can be defined via physical distance, social distance or phenotypic distance^20^,^22^,^31^,^33^,^34^,^35^,^36^,^37^,^38^,^39^.

Although population structure can affect the outcome of any evolutionary game^18^,^20^,^40^,^41^,^42^,^43^,^44^,^45^,^46^,^47^, many studies focus on evolution of cooperation^18^,^19^,^37^,^48^,^49^,^50^,^51^,^52^,^53^,^54^,^55^,^56^. A typical, crucial question is: what is the minimum benefit-to-cost ratio needed for spatial selection to favor cooperation over defection^10^,^39^,^55^,^57^,^58^,^59^,^60^,^61^? 

Recently, it was reported that “shift” updating is a strong promoter of cooperation in a one dimensional setting^61^. An individual is chosen for reproduction at random but proportional to payoff. The offspring is placed next to it. A random individual dies. The individuals rearrange (shift) on the cycle until each position is again occupied by a single individual. For large population size, cooperation is favored over defection if the benefit-to-cost ratio exceeds one. This is the minimum possible critical benefit-to-cost ratio. This condition always holds if cooperation is beneficial.

Attempts to generalize the “shift” updating rule to higher dimensions, while maintaining its strong effect, have failed, because the rearrangement of individuals in higher dimensions destroys the clusters of cooperators^61^. Here we introduce a repulsive force between cells of different types. Then the actual path of rearrangement in two dimensions is calculated taking into account the repulsive force and minimizing energy. This very natural, physical process leads to a strong effect of promoting evolution of cooperation.

## Evolutionary process: The Generic Shift Rule Model

We consider a heterogeneous population of cells that is structured in a two-dimensional grid as the most well-studied case of regular graphs in two dimensions^7^,^8^,^9^,^22^,^49^,^50^,^51^,^55^. Individual cells reproduce and die: a cell death leaves a vacant position of low pressure in the grid, whereas a cell reproduction generates a duplicate cell in that position, and induces higher pressure. This pressure difference in the grid is resolved by shifting the cells from the high to the low pressure position, along some path. The path to be chosen is determined by forces acting between the cells. Afterwards, the population is again spread out evenly on the grid, with one cell in each position.

### Evolutionary process on a grid

We consider a discrete-time evolutionary process on a population of *N* = *n*^2^ individuals, each occupying one position in a *n* × *n* grid. Each individual at position (*i*, *j*), for 0 ≤ *i*, *j* ≤ *n* − 1, is of one type, either A or B, and has four neighbors at positions (*i*′, *j*′) such that |*i* − *i*′| (mod *n* − 2) + |*j* − *j*′| (mod *n* − 2) = 1. (i.e., the grid wraps around and forms a torus).

The types of an individual and its neighbors determine its *fecundity* (reproductive rate). At every time point, one individual is chosen uniformly at random to die, and one individual is chosen to reproduce randomly and proportionally to its fecundity. These two events are independent, and might result in the same individual. Let (*i*_1_, *j*_1_) and (*i*_2_, *j*_2_) be the positions on the grid of the death and the reproduction events, respectively. The population is updated by the following generic *shift rule*:

1. Choose a path 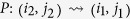.
2. Shift the individuals that appear in the intermediate positions of *P* one step forward in the direction of *P*. Now the position (*i*_1_, *j*_1_) is occupied by a neighbor of the dead individual, and no individual occupies the neighboring position of (*i*_2_, *j*_2_) in *P*. Finally, place a copy of the reproducing individual in the vacant position (i.e., the neighbor of (*i*_2_, *j*_2_) in *P*).

**Figure 1:**
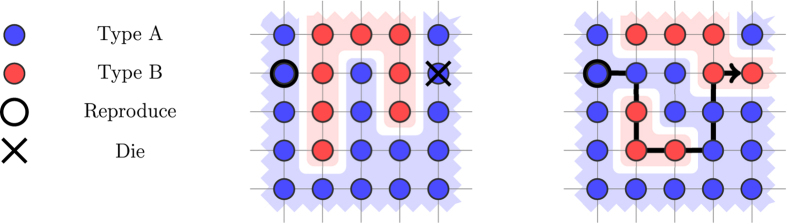
Illustration of the generic shift rule on a two-dimensional grid. A death event at position (4, 3) (counting from zero, with (0, 0) being the lower left corner) creates a vacancy in the population structure. A birth event at position (0, 3) creates a new cell. Finally, all cells are shifted along a path 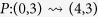, so that afterwards, the population size remains unchanged and all cells are evenly spaced out.

Figure 1 illustrates one step of this process. We will later discuss two different ways of choosing the path *P*, i.e., give two instances of the generic shift rule. We now consider the notion of games to assign fecundities to individuals.

### Discrete games

At each step of the evolutionary process, each individual is chosen for reproduction with probability that is proportional to its fecundity. This fecundity is not static, but determined by the individual’s interactions with each of its neighbors, via a matrix game^4^,^8^. In particular, each such interaction contributes the payoff at a position according to the matrix (1), where the row is the type of the individual, and the column is the type of the neighbor.


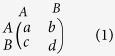


Our work focuses on the additive Prisoner’s Dilemma, where the payoffs are given by matrix (2). Here we consider a fixed cost *c* = 1 for offering cooperation, and a varying benefit *b* for receiving cooperation.


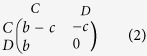


The average payoff *π* received by the individual from interactions with neighbors is mapped to fecundity as 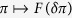 where *δ* > 0 is a real number that quantifies the intensity of selection, and *F* is a positive, differentiable, increasing function. There exist various choices for the function *F* in the literature^61^,^62^. In this work, we focus on the frequently used linear map, with 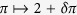
39,60,63. We will consider both *strong selection* where *δ* = 1, and the *limit of weak selection* where *δ* ≪ 1 (or *δ* → 0). In the strong selection regime, this is equivalent to the more commonly used map 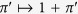, in which the average utility *π*′ comes from the modified Prisoner’s Dilemma of matrix (3), where every entry has been increased by *c* = 1 to ensure that all individuals always have non-zero fecundity.


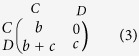


## Fixation probability

We quantify the evolutionary success of each type of individuals by means of their ability to invade populations of the opposite type. In particular, we are interested in the probability *ρ*_*C*_ that a single cooperator mutant arising in a population of defectors will eventually take over the population. Similarly, the fixation probability of a single defector mutant in a population of cooperators is denoted by *ρ*_*D*_. As both fixation probabilities decrease with the population size *N*, we are interested in the relative fixation probability 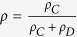, and particularly in cases where cooperation is favored, i.e., *ρ* ≥ 0.5^39^,^56^,^61^.

### Fixation probability in other models

**Figure 2:**
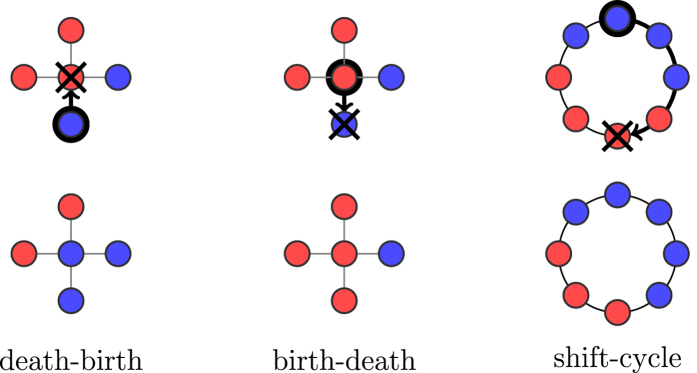
Three well-studied update rules on graphs. *Left:* In the death-birth rule, one individual is chosen uniformly at random to die and leaves a vacant position. Then, a neighbor is chosen to reproduce with probability proportional to its fecundity. The reproducing individual places a copy of itself in the vacant position. *Center:* In the birth-death rule, one individual is chosen to reproduce with probability proportional to its fecundity. Then, a neighbor is chosen uniformly at random to die, and leaves a vacant position, which is filled with a copy of the reproducing individual. *Right:* In the shift rule on a cycle, the population is arranged on a cycle, such that each individual has two neighbors. This forms a 1-dimensional grid. One individual is chosen uniformly at random to die, and one to reproduce with probability proportional to its fecundity. The reproducing individual places a copy of itself in a neighboring position, and the rest of the population shifts in one direction to fill the vacant position.

The current work lies in the line of research for natural evolutionary processes that lead to rise of cooperation when the benefit of receiving cooperation is not too large as compared to the cost of offering it (i.e., *b*/*c* is small)^10^,^39^,^55^,^57^,^58^,^59^,^60^,^61^. For the special case where individual payoffs are derived from the Prisoner’s Dilemma game^3^, has used the term *critical benefit-to-cost ratio* (*b*/*c*)^*^ to refer to the least value of the *b*/*c* ratio which favors cooperation. Some of the most well-studied processes (or, so called, “update rules”) on graphs are the death-birth rule and the birth-death rule of^39^, and the shift rule on a cycle of^61^. For an illustration, see Fig. 2.

As shown by^39^, the birth-death rule does not promote cooperation (i.e., *ρ* < 0.5) regardless of the benefit-to-cost ratio. To explain this intuitively, we first observe that the only relevant birth-death events occur along an edge connecting individuals of different types. As stated by^39^, on average, the number of cooperator neighbors is the same for the two individuals. Thus the benefit received from the neighbors is, on average, the same for each individual. However, as long as *c* > 0, the cooperating individual suffers a cost for offering cooperation, and thus has less fecundity than the defector. Therefore, when the birth-death event occurs in the edge, the defector has higher chance of reproducing than the cooperator.

In contrast, in the death-birth rule cooperation is favored in the limit of weak selection (*δ* ≪ 1), as long as (*b*/*c*) > *κ*, where *κ* is the average number of neighbors of each individual. Hence here the critical ratio is (*b*/*c*)^*^ = *κ*, and *κ* = 4 for two-dimensional grids. The intuitive description behind this threshold is as follows. Consider a vacant position created by a death event, and a pair of individuals of different types that compete for placing a copy. The pair approximation of^39^ shows that for the death-birth rule, the cooperating individual has on average one more cooperating neighbor, among its *κ* − 1 neighbors, than the defector. Hence, the fecundity of the cooperator is larger than that of the defector as long as the additional benefit from the extra cooperation balances the total cost from offering cooperation to *κ* neighbors, i.e., *b* > *κc*.

Given the result for the death-birth rule, an important question is whether there exist other update rules which favor cooperation for *b*/*c* < *κ*. A positive answer was provided for one-dimensional population structure in^61^. A cycle is a special case of the torus (i.e., it is one-dimensional torus), and the shift rule on a cycle is an instance of choosing the path *P* in the generic shift rule. The shift rule on a cycle of^61^ favors cooperation as long as *b* > *c*. Note that in a cycle *κ* = 2, and thus the result of^61^ is a witness of an update rule with critical ratio (*b*/*c*)^*^ = 1 < *κ*. In contrast to the *local*^64^ birth-death and death-birth rules, the shift rule on a cycle is a *global* update rule, which means that all the death and reproduce events depend on the relative fecundity of each individual. When an individual dies, the whole population of cooperators competes with the population of defectors for filling the vacancy. On a cycle, we always have one cluster of each type, and except for the two cooperators and defectors at the boundary we have the following: the payoff of the defectors is 0, whereas as long as *b* > *c*, the cooperators have positive payoff. The defectors at the boundary have payoff *b* each, and hence the total payoff of all defectors is 2*b* (i.e., constant and irrespective of the population size), whereas each cooperator other than the two at boundary has positive payoff. Thus for sufficiently large populations, the total fecundity of the cooperators is larger than that of the defectors, and reproduction is biased towards cooperators.

## Results

The fundamental question for the two-dimensional population structure is again whether there can be instances of the generic shift rule where the critical ratio is less than *κ*, as compared to the death-birth process. For two-dimensional population structure such as a grid we have *κ* = 4. Given the fundamental question and its affirmative answer in the one-dimensional grid, a natural question is whether the shift rule on a cycle can be extended to two dimensions. This question has been explored, but without success in^61^. In particular, the shift rule on a cycle was lifted to the two-dimensional case, by choosing the path *P* from the reproduction to the death position randomly among the shortest paths wrt the number of hops (i.e., the number of steps). However, this rule does not favor cooperation even with *b*/*c* = 4. The authors in^61^ identified that the key difference between one and two dimensions is that in the former case, the two types of individuals always form one cluster each, whereas in the latter case, shifting paths between distant events would regularly disrupt clusters. This lead to longer boundaries between cooperators and defectors, which defectors would exploit to increase their payoff, and thus fecundity.

The failure of the natural generalization of the shift rule on a cycle brings us to the following questions: does there exist any natural instance of the generic-shift rule in the torus that (i) has critical ratio (*b*/*c*)^*^ < 4 under weak selection (thus favoring cooperation more strongly than the death-birth rule) and (ii) has critical ratio (*b*/*c*)^*^ smaller than the generalization of the shift rule on a cycle in two dimensions. We will present affirmative answers to these questions. First, we introduce two instances of the generic shift rule for choosing the path *P*.

### Two instances of the generic shift rule

We consider that between every individual and neighbor there exists a *repulsion force* measured as a real value. This force is a measure of the resistance when a copy of the individual tries to occupy the position of the neighbor. Note that this repulsion force is specific to the pair under consideration, and can generally differ among different pairs.

**Figure 3:**
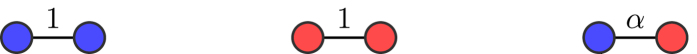
The repulsion force between neighbors has value normalized to 1 for a pair of same-type individuals, and some real value *α* ≥ 1 for a pair of different-type individuals.

In this work we consider two natural ways for obtaining the paths 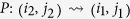, which lead to two instantiations of the generic shift rule. First, we fix a symmetric repulsion force between neighbors that has value (i) *α* ≥ 1 between different-type individuals, and (ii) fixed to 1 between same-type individuals (Fig. 3). This captures *affinity* between neighbors. Neighbors of the same type are linked with a large affinity, and the repulsion force along their edge is small, i.e., 1. In contrast, neighbors of different types share a smaller affinity, and the repulsion force is generally larger, i.e., *α* ≥ 1.

Given a path *P*, we denote by *α*(*P*) the sum of repulsion forces along the edges of *P*. Once the death event has taken place, a vacancy is left in (*i*_1_, *j*_1_). Every individual in position (*i*, *j*) is associated with a *resistance value* 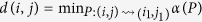 which is the smallest sum of the resistance forces that the individual in position (*i*, *j*) feels along a path to the vacancy in (*i*_1_, *j*_1_). We consider the following two rules for choosing the path 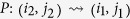 from reproduction to death.

**Figure 4:**
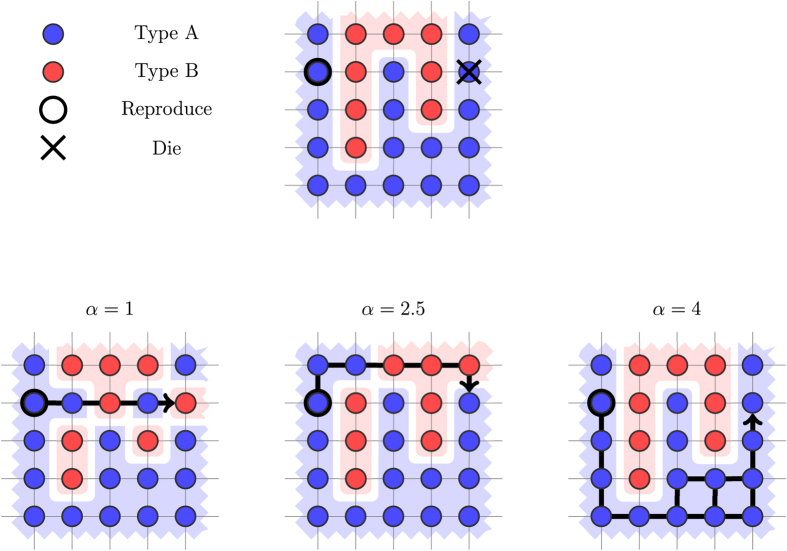
Illustration of the LR shift rule. The least-resistance path from the reproducing to the dying cell differs as the repulsion force *α* increases. Initially, we have one cluster of Type B individuals. When we update for *α* = 1, the result is four clusters of Type B individuals. When we update for *α* = 2.5, the result is two clusters of Type B individuals. When we update for *α* = 4, the grid remains unchanged, and we have only one cluster.

**Figure 5:**
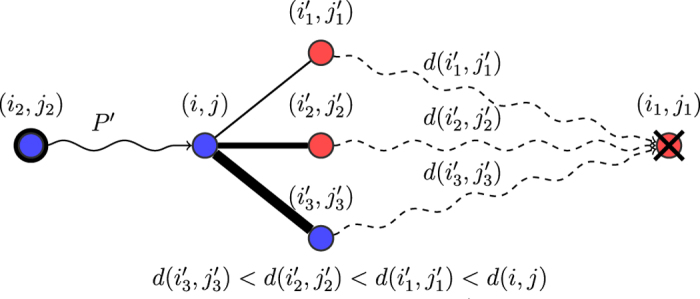
Illustration of the LNR shift rule. The path 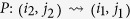 from reproduction to death is formed dynamically. Given a current prefix 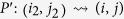, a neighbor (*i*′, *j*′) of (*i*, *j*) is likely to extend *P*′ with probability proportional to 1/*d*(*i*′, *j*′), as long as *d*(*i*′, *j*′) < *d*(*i*, *j*). The process repeats until (*i*_1_, *j*_1_) is reached.

1. *Least-resistance rule (LR).* In the LR rule, informally, the path *P* is chosen uniformly at random among the least-resistance paths. Formally, let *d* = *d*(*i*_2_, *j*_2_) denote the resistance value of (*i*_2_, *j*_2_). Let 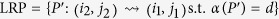 be the set of paths *P*′ from (*i*_2_, *j*_2_) to (*i*_1_, *j*_1_) that achieve the resistance value. In other words LRP represents the set of all least-resistance paths from (*i*_2_, *j*_2_) to (*i*_1_, *j*_1_). The path *P* is then chosen uniformly at random from the set LRP. Intuitively, this captures a least-effort principle, where the reproducing individual will fill the hole while spending as little energy as possible. Figure 4 provides an illustration of how the shift dynamics are affected by choosing different values *α* for the repulsion force between heterogeneous pairs in the LR rule.
2. *Least neighbor-resistance rule (LNR).* In the LNR rule, informally, the path *P* is chosen by decreasing proportionally to the resistance to (*i*_1_, *j*_1_) in each step. Formally, for each position *v* = (*i*, *j*) in the grid, let Nh(*v*) be the set of its neighbors, and NhR(*v*) = {*v*′ ∈ Nh(*v*):*d*(*v*′) < *d*(*v*)} denote the neighbors with strictly smaller resistance value. Informally, every such neighbor is a candidate for being chosen as the next position in the path, and the probability to be chosen is inversely proportional to the resistance values. Formally, for a position *v* and a neighbor *v*′ in NhR(*v*), let 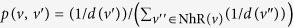, denote the probability to choose *v*′ as the next position from *v*. In other words, at position *v* the next vertex is chosen inversely proportional to the resistance values to decrease the resistance (i.e., the lower the resistance of a neighbor, the higher is the probability to be chosen). The path *P* is chosen using the following stochastic process: the starting position is (*i*_2_, *j*_2_), and at each step if the current position is *v*, then the next position *v*′ ∈ NhR(*v*) is chosen with probability *p*(*v*, *v*′), until (*i*_1_, *j*_1_) is reached. This rule also follows a least-effort principle, but the choice is now made locally at each step rather than choosing among the least-resistance paths. Figure 5 illustrates the above process.

#### Important features

We discuss three important aspects of the introduced instances of the shift rule. First, the least-resistance principle is a natural one, which captures affinity between neighbors. The LR rule chooses uniformly among the global least-resistance choices; whereas the LNR rule follows a stepwise reduction in the resistance. Second, these shift rules are very simple, as they are based on just one parameter *α*. Finally, many rules can be obtained as special case of the LR and LNR rules, e.g., for *α* = 1, we have that both LR and LNR coincide with the natural generalization of the shift rule on a cycle.

### Strong selection

In this section we present results for strong selection, where we compare the LR and the LNR rule for various values *α* against the death-birth rule, and the same rules for the specific value of *α* = 1, which is the generalization of the shift rule on a cycle.

**Figure 6:**
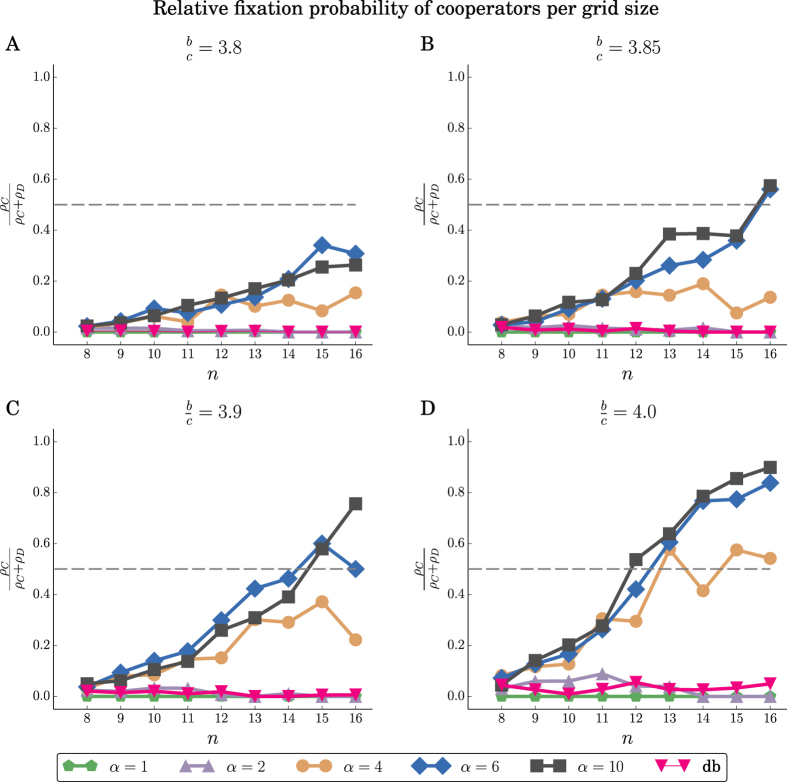
LR rule. Relative fixation probability 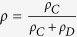 of cooperators in *n* × *n* grids, for different values of *n*, and benefit-to-cost ratios *b*/*c*. The fixation probabilities *ρ*_*C*_ (resp. *ρ*_*D*_) correspond to a single cooperator (resp. defector) mutant in a population of defectors (resp. cooperators). The db denotes the death-birth process run for the same parameters. As *b*/*c* and *α* increase, *ρ* also increases and exceeds 0.5, and cooperation is favored. The fixation probabilities are averages over 10^4^ replicates.

**Figure 7:**
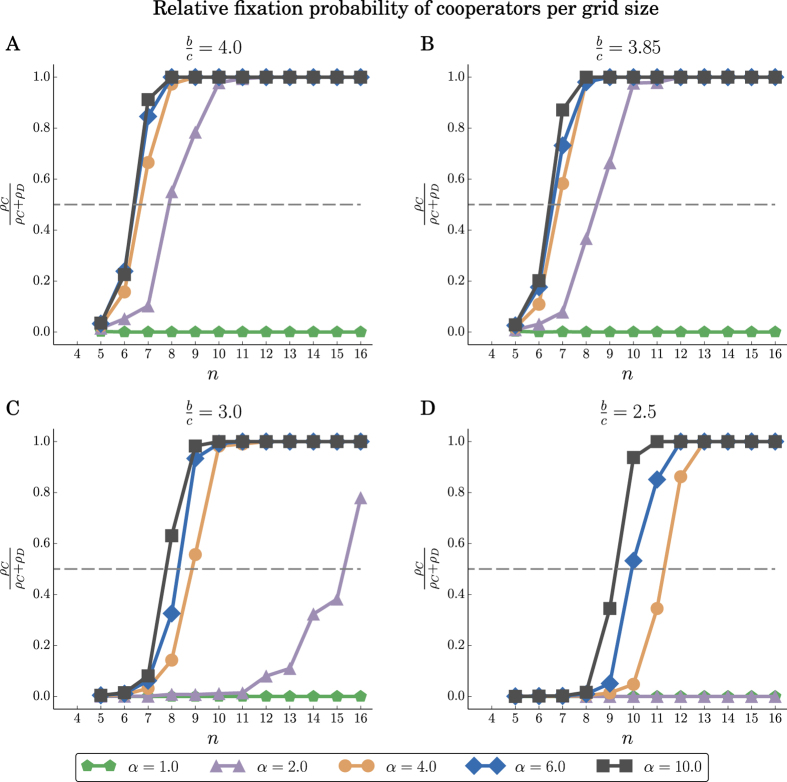
LNR rule. Relative fixation probability 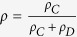 of cooperators in *n* × *n* grids, for different values of *n*, and benefit-to-cost ratios *b*/*c*. The fixation probabilities *ρ*_*C*_ (resp. *ρ*_*D*_) correspond to a single cooperator (resp. defector) mutant in a population of defectors (resp. cooperators). db denotes the death-birth process run for the same parameters. As *b*/*c* and *α* increase, *ρ* also increases and exceeds 0.5, and cooperation is favored. The fixation probabilities are averages over 10^4^ replicates.

We have used simulations to evaluate the success of cooperation under the two instantiations of the shift rule on 2-dimensional grids. The simulations cover various grid sizes, benefit-to-cost ratios (with fixed *c* = 1), and values of the repulsion force *α*. The results for the LR rule are shown in Fig. 6. We find that for larger values of *α*, cooperation is favored (*ρ*_*C*_ > *ρ*_*D*_) even for (*b*/*c*) < 4, which is the required threshold for the death-birth rule in case of 2-dimensional grids. In contrast, in such a strong selection setting (*δ* = 1) and for small/moderate population sizes *N*, the death-birth rule or *α* = 1 favors defection (see Fig. 6). The results for the LNR rule are shown in Fig. 7. Here cooperation is favored for much smaller *b*/*c* ratios and values *α* of the repulsion force.

**Figure 8:**
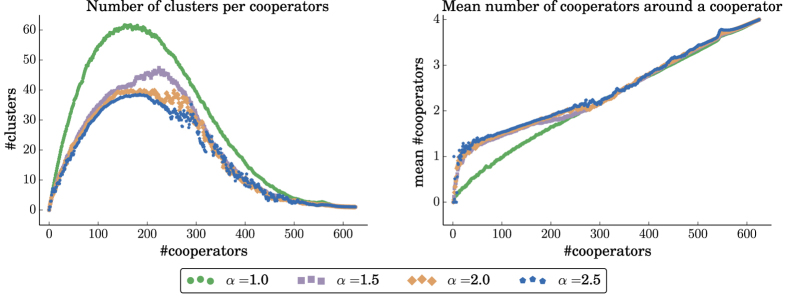
Statistics on a 25 × 25 grid of cooperators with an initial cluster 9 × 9 of defector mutants, for *b* = 4 and different values of the repulsion force *α*. Data points are averages over 200 replicates. *Left:* The increase in the number of cooperators in the grid leads to the formation of cooperator clusters. Higher values of the repulsion force lead to fewer, larger clusters. *Right:* The average number of cooperators around a cooperator as the number of cooperators increases. Higher values of the repulsion force lead, on average, to more cooperators per cooperator. The difference is more pronounced when the cooperators are the minority in the population (i.e., left part of the figures).

**Figure 9:**
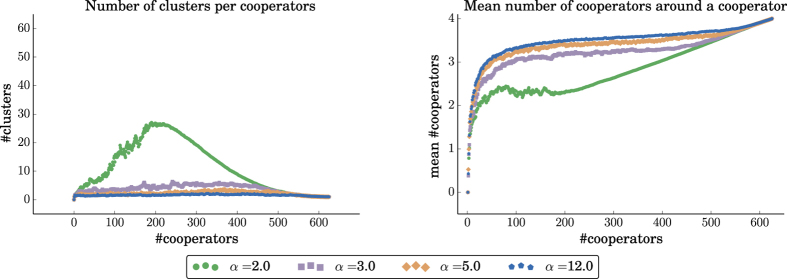
Statistics on a 25 × 25 grid of cooperators with an initial cluster 2 × 2 of cooperator mutants, for *b* = 3 and different values of the repulsion force *α*. Data points are averages over 200 replicates. *Left:* The increase in the number of cooperators in the grid leads to the formation of cooperator clusters. Higher values of the repulsion force lead to fewer, larger clusters. *Right:* The average number of cooperators around a cooperator as the number of cooperators increases. Higher values of the repulsion force lead, on average, to more cooperators per cooperator. The difference is more pronounced when the cooperators are the minority in the population (i.e., left part of the figures).

To explain the evolutionary advantage of cooperators for larger values of the repulsion force, we have peeked into the structure of the population throughout the evolutionary process, and in particular, in the number of cooperator clusters, and on the average number of cooperating neighbors that a cooperator has. We find that as *α* increases, the population of cooperators is clustered in a small number of large clusters. On average, this leads to each cooperator having more cooperating neighbors (see Figs 8 and 9), and thus increases the fecundity of the population of cooperators. These results also explain the significant advantage of the LNR over the LR rule, as under the LNR rule, the cooperators are grouped in fewer, larger clusters, and thus on average a cooperator has many more cooperating neighbors.

As discussed earlier, the shift rule on a grid reduces to the shift rule on a cycle if we consider 1-dimensional grids. This is because in the case of a cycle, the path from the reproducing to the dying individual does not affect the state of the population. However, in the opposite direction, there are several update rules that generalize the shift rule from a cycle to a 2-dimensional grid. The early efforts of^61^ for shift updates on a grid lead to rules that fail to promote cooperation, as in higher dimensions clusters of cooperators were easily disrupted by invading defectors. Indeed, same-type individuals are not clustered together, and the state of the population is not described by a single parameter (e.g., the number of cooperators), as in the case of the cycle. Here we find that by introducing repulsion force between different-type individuals, cooperation is favored, for small benefit-to-cost ratios.

### Computational bottlenecks

Determining the relative fixation probability 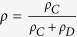 via computer simulations is a computationally expensive process. Observe that the fixation probabilities *ρ*_*C*_ and *ρ*_*D*_ are small, even for small grid sizes, as they represent the probability of fixation of a single individual in a population of different-type individuals. As *ρ* is a fraction of small numbers, it is unstable in small perturbations of *ρ*_*C*_ and *ρ*_*D*_. Thus a robust approximation of *ρ* requires estimating *ρ*_*C*_ and *ρ*_*D*_ to a significant precision, via many replicates (different runs) of the simulated process. In our simulation we ran 10^4^ replicates. For example, in grid 16 × 16, the population size is 256, and the time to fixation is around 10^3^ steps. Each step requires a shortest-path computation, which for graphs of size *N* requires *N*⋅log*N* time, (i.e., in our case around 1.4 × 10^3^ operations) (for further details see SI). Hence for each initial condition we require 1.4 × 10^10^ operations, and since we have two initial conditions (a single cooperator and a single defector to start with), the total number of computational steps is 2.8 × 10^10^. Since we have many different *α* values and many different grid sizes, the total computational steps required were around 5 × 10^11^. Hence computer simulations to obtain results for larger grid sizes is infeasible in reasonable time.

### Weak selection

We now consider the weak selection regime, where *δ* ≪ 1. For weak selection, estimating the fixation probabilities directly from simulated runs is not possible, as the payoffs of individuals impact the evolution of the population only in the long run. Hence to understand the weak selection scenarios we need to consider mathematical results, which we discuss next.

The critical benefit-to-cost ratio for favoring cooperation in structured populations in the weak selection regime is characterized by the *structure coefficient σ*^43^,^64^. In particular, we consider a mutation rate *μ* ≤ 1, such that every reproducing individual has probability *μ* to produce an offspring of the different type. Moreover, to keep the effect of mutation minimal, we consider the low-mutation case, where given a population of size *N* we have *Nμ* ≪ 1. Now neither cooperators nor defectors get fixed, but instead they coexist in different proportions in the population, and the evolutionary success of either type is quantified by its abundance in the steady state. It has been shown in^43^ that there exists a parameter *σ* (structure coefficient) such that the critical benefit-to-cost ratio for which cooperators are more abundant than defectors is


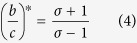


when payoffs are received from individual interactions according to matrix (2). The structure coefficient *σ* can depend on the structure of the population, the update rule, the mutation rate and other parameters, but not on the payoffs *b* and *c*. Hence Eq. (4) allows to characterize the relative abundance of cooperators wrt two orthogonal parts; the benefit-to-cost ratio captures the contribution of the parameters of the game, and *σ* captures the contribution of the evolutionary process. As shown in^64^, there is a simple formula to calculate *σ* for the large class of evolutionary processes that satisfy the following conditions:

1. *global updating*, i.e., all individuals compete uniformly for reproduction and
2. *constant birth or death rate*, i.e., the individual payoffs do not affect both births and deaths.

In this case, we can write


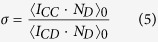


where *I*_*XY*_ denotes the number of interactions between type *X* and type *Y* individuals in a state, *N*_*X*_ denotes the number of type *X* individuals, and 〈⋅〉_0_ denotes averaging over all possible states of the stochastic process under neutral drift (*δ* = 0). The evolutionary process introduced in this paper (i.e., the LR and LNR rule) satisfies (i) and (ii), and thus Eq. (5) is applicable.

Even though Eq. (5) is applicable, it is hard to compute analytically. However, the equation suggests a way to approximate *σ*, by simulating the evolutionary process under neutral drift sufficiently long. In our results, we simulated the evolutionary processes under neutral drift, from various initial conditions, for sufficiently long until they all reasonably converged to produce the same result. Here we report on approximations of *σ* and the critical benefit-to-cost ratio obtained from them for the following three update rules.

1. LNR with repulsion force *α* = ∞,
2. LR with repulsion force *α* = ∞,
3. LNR, LR with repulsion force *α* = 1 (in this case the two rules coincide).

Our results are shown in Table 1. For each entry in the table we ran the computation for various initial conditions, for 10^9^ steps, in parallel, which took around 4 weeks. We report these results, which at best is an approximation of the critical benefit-to-cost ratio, and the original benefit-to-cost ratio might differ by a small amount. While for the LNR rule with *α* = ∞ the critical benefit-to-cost ratio is well below 4, for the LR rule with *α* = ∞ it is around 4, whereas with *α* = 1, it is well above 4. Also note that for the death-birth rule the critical benefit-to-cost ratio of 4 for grids is only in limit the large populations, whereas our results are for population size up to 400.

## Discussion

We now discuss some important aspects of our results. First, we report success on designing natural rules to favor cooperation in a two-dimensional grid. Second, our result also shows some robustness in the following sense. Our key principle is introducing a single parameter *α* for the repulsion force that captures assortment between individuals of the same type. We show that two different simple rules (LR and LNR) based on this principle lead to critical benefit-to-cost ratio (*b*/*c*)^*^ < 4, which is the the critical ratio for the death-birth rule. Note that the critical benefit-to-cost ratio of 4 for the death-birth rule in two-dimensional grid is only for population of large sizes, whereas for our rules even for moderate population size (such as 400) both LR and LNR have critical benefit-to-cost ratio strictly below 4 (and for LNR rule this holds for even population size of 100). Intuitively, the repulsion force favors update paths that cross fewer clusters, and thus leading to fewer disruptions of the cooperator clusters, which is a crucial ingredient for the rise of cooperation^2^,^3^,^10^. While the LNR rule works better than the LR rule, the fact two different rules can be developed based on a single principle, shows that the principle we propose is a robust one.

Animated illustrations of the various evolutionary processes discussed (namely, death-birth, LR and LNR) and different values of selection intensity and repulsion force can be found in http://pub.ist.ac.at/~pavlogiannis/grid/.

## Conclusion

We have studied evolutionary game dynamics on a two-dimensional grid. While the shift-update rule^61^ is a strong promoter of cooperation in one dimension, all previous generalizations of the rule to higher dimensions have failed to promote cooperation. Here we introduce two natural variants of shift updating, LR and LNR, based on a repulsion force of cells of different type and an energy-minimization principle. We show that those rules promote cooperation on the two-dimensional grid for strong and for weak selection. An interesting direction of future work is to consider those rules for higher dimensions and other population structures. A natural extension is also to study how rules could generate semi fluid population structures with a tunable level of viscosity.

## Additional Information

**How to cite this article**: Pavlogiannis, A. *et al.* Cellular cooperation with shift updating and repulsion. *Sci. Rep.* **5**, 17147; doi: 10.1038/srep17147 (2015).

## Figures and Tables

**Table 1:** The critical ratio (*b*/*c*)^*^ approximated for different grid length sizes *n* and update rules.

	LNR (*α* = ∞)	LR (*α* = ∞)	LNR, LR (*α* = 1)
*n* = 10	2.0	4.0	7.0
*n* = 14	1.6	4.0	7.1
*n* = 18	1.4	3.9	9.2
*n* = 20	1.3	3.9	10.2

We consider mutation rate *μ* = 10^−3^ to ensure *Nμ* < 1.
